# Impact of ALK Inhibitors in Patients With *ALK*-Rearranged Nonlung Solid Tumors

**DOI:** 10.1200/PO.20.00383

**Published:** 2021-05-03

**Authors:** Yuki Takeyasu, Hitomi S. Okuma, Yuki Kojima, Tadaaki Nishikawa, Maki Tanioka, Kazuki Sudo, Tatsunori Shimoi, Emi Noguchi, Ayumu Arakawa, Taisuke Mori, Kuniko Sunami, Takashi Kubo, Takashi Kohno, Yoshida Akihiko, Noboru Yamamoto, Kan Yonemori

**Affiliations:** ^1^ Department of Breast and Medical Oncology, National Cancer Center Hospital, Tokyo, Japan; ^2^ Course of Advanced Clinical Research of Cancer, Juntendo University Graduate School of Medicine, Tokyo, Japan; ^3^ Clinical Research Support Office, National Cancer Center Hospital, Tokyo, Japan; ^4^ Department of Pediatric Oncology, National Cancer Center Hospital, Tokyo, Japan; ^5^ Department of Diagnostic Pathology, National Cancer Center Hospital, Tokyo, Japan; ^6^ Department of Laboratory Medicine, National Cancer Center Hospital, Tokyo, Japan; ^7^ Division of Translational Genomics, Exploratory Oncology Research and Clinical Trial Center, National Cancer Center, Tokyo, Japan; ^8^ Department of Clinical Genomics, National Cancer Center Research Institute, Tokyo, Japan; ^9^ Division of Genome Biology, National Cancer Center Research Institute, Tokyo, Japan; ^10^ Rare Cancer Center, National Cancer Center Hospital, Tokyo, Japan; ^11^ Department of Experimental Therapeutics, National Cancer Center Hospital, Tokyo, Japan

## Abstract

**PURPOSE:**

Anaplastic lymphoma kinase (*ALK*) rearrangement is a well-known driver oncogene in non–small-cell lung cancer and has also been identified in other types of tumors. However, there is limited evidence on the clinical response to ALK tyrosine kinase inhibitors (TKIs), such as alectinib and crizotinib, in rare tumors with ALK fusion. We evaluated the therapeutic effect of ALK-TKIs in rare *ALK*-rearranged tumors.

**PATIENTS AND METHODS:**

Between April 2012 and April 2019, clinical outcomes and characteristics of patients with *ALK*-rearranged nonlung solid tumors who received ALK-TKIs (alectinib and/or crizotinib) outside of clinical trials were reviewed. Expression and/or rearrangement of ALK was evaluated by immunohistochemistry, fluorescence in situ hybridization, and next-generation sequencing. The tumor response was assessed according to RECIST (version 1.1). Progression-free survival was estimated from initial ALK-TKI initiation until progression.

**RESULTS:**

We identified seven patients (inflammatory myofibroblastic tumors, n = 3; ALK-positive histiocytosis, n = 1; histiocytic sarcoma, n = 1; osteosarcoma, n = 1; and parotid adenocarcinoma, n = 1), with a median age of 17 years. Two rare *ALK* fusions, namely, *CTNNA1-AL*K and *ITSN2-ALK*, were identified. As initial ALK-TKI therapy, five patients received alectinib and two received crizotinib. The objective response rate for the initial ALK-TKI therapy was 85.7% (95% CI, 44 to 97), including two patients who received alectinib and achieved complete response. The median progression-free survival was 8.1 months (range, 1.7 to not estimable). There were no treatment interruptions or dose reductions because of adverse events caused by alectinib.

**CONCLUSION:**

This study highlights the potential benefit of ALK-TKIs, especially alectinib, in patients with *ALK*-rearranged nonlung solid tumors.

## BACKGROUND

Anaplastic lymphoma kinase (*ALK*) rearrangement was first discovered as a potential actionable therapeutic oncogenic gene aberration in anaplastic large-cell lymphoma (ALCL).^1^ The next disease linked to *ALK* was inflammatory myofibroblastic tumor (IMT), characterized by increased expression of the ALK protein and a rearranged *ALK* locus in a subset of cases.^2,3^ Later, there were advances in *ALK*-targeted therapy because of the discovery of the *EML4-ALK* rearrangement in non–small-cell lung cancer (NSCLC),^4^ which led to the discovery of *ALK* alterations in other solid tumors such as neuroblastomas, rhabdomyosarcomas, and anaplastic thyroid cancers.^5-7^ Activation of the *ALK* gene can occur by rearrangements with partner genes, point mutations, or amplification. Ever since the discovery of the oncogenic role, *ALK* alterations, mainly rearrangements, have been a target for developing therapies, and Hiroyuki Mano^8^ proposed the collective name ALKoma to refer to tumors that develop because of *ALK* functioning abnormally as an oncogene.

**CONTEXT**

**Key Objective**
There is limited evidence on the clinical efficacy of anaplastic lymphoma kinase (ALK) tyrosine kinase inhibitors (ALK-TKIs) in nonlung solid tumors with *ALK* rearrangements. This single institutional study aimed to evaluate the efficacy and tolerability of ALK-TKIs in a group of patients with *ALK*-rearranged rare solid tumor.
**Knowledge Generated**
Alectinib showed dramatic response for patients with *ALK*-rearranged nonlung solid tumors, and furthermore, sequential ALK-TKI therapy was acceptable for some patients. A patient with parotid tumor with *CTNNA1-ALK* rearrangement derived clinical benefit from alectinib.
**Relevance**
Our data revealed that *ALK* rearrangements are found in rare solid tumors and result in clinical benefit when treated with ALK-TKIs. This leads to a rationale for clinical trials targeting *ALK*-rearranged nonlung solid tumors to promote personalized medicine.

The first-generation ALK-tyrosine kinase inhibitor (TKI) to be approved following clinical trials was crizotinib, which also acts as a mesenchymal epithelial transition factor and receptor tyrosine kinase-1 kinase inhibitor. It showed a dramatic effect in ALK-positive solid tumors and ALCLs.^9,10^ A second-generation ALK inhibitor, alectinib, was subsequently approved and showed a higher response rate than crizotinib with minimal toxicity in patients with *ALK*-rearranged metastatic NSCLC.^11^ Currently, ceritinib, brigatinib, and lorlatinib are approved in the clinical setting for *ALK*-rearranged NSCLC.^12-14^

In ALKomas other than NSCLC, a small sample size of phase II studies showed that crizotinib demonstrated antitumor efficacy and achieved a durable response, as anticipated.^15^ However, there are limited reports on other ALK-positive solid tumors, and the efficacy and safety of alectinib for these tumors have only been described as case reports.^16-19^ Thus, in this case series, we summarize the efficacy and tolerability of ALK-TKIs (alectinib and crizotinib) in patients with ALKomas.

## PATIENTS AND METHODS

We retrospectively reviewed patients with *ALK*-rearranged nonlung solid tumors who received alectinib and crizotinib outside of clinical trials at the National Cancer Center Hospital between April 2012 and April 2019. Patient data were retrieved from electronic medical records. Alectinib and crizotinib were administered at the doses approved for NSCLC in Japan: 300 mg twice daily and 250 mg twice daily, respectively. Expression of ALK and/or rearrangement of *ALK* was evaluated by immunohistochemistry (IHC) (5A4, Abcam, Cambridge, UK, or ALK1, DAKO, Glostrup, Denmark), fluorescence in situ hybridization (FISH) using a break-apart probe (Vysis ALK Break Apart FISH Probe Kit, Abbott Molecular, Abbott Park, IL), or next-generation sequencing (NGS) using NCC Oncopanel v4.0, which detects gene rearrangements, base substitutions, short insertions or deletions, and copy number alterations in 114 genes.^20^ The institutional ethics committee of the National Cancer Center Hospital approved this study (#2016-086). We also obtained documented informed consent from each patient before treatment. The response to ALK-TKI was assessed by two independent oncologists according to version 1.1 of the RECIST.^21^ The response rate (ie, the proportion of patients with complete response (CR) or partial response [PR]) was calculated, and its 95% CI was estimated based on the Clopper-Pearson method. Time-to-event end points were summarized using the Kaplan-Meier method. Data were analyzed using JMP Pro version 13.0.0 (SAS Institute).

## RESULTS

Among the patients treated with an ALK-TKI outside of a clinical trial during the study period, seven had nonlung solid tumors. Initial ALK-TKI treatment consisted of alectinib in five patients and crizotinib in two patients. Patient characteristics are shown in Table 1. The median follow-up time was 15.0 months.

**TABLE 1.**
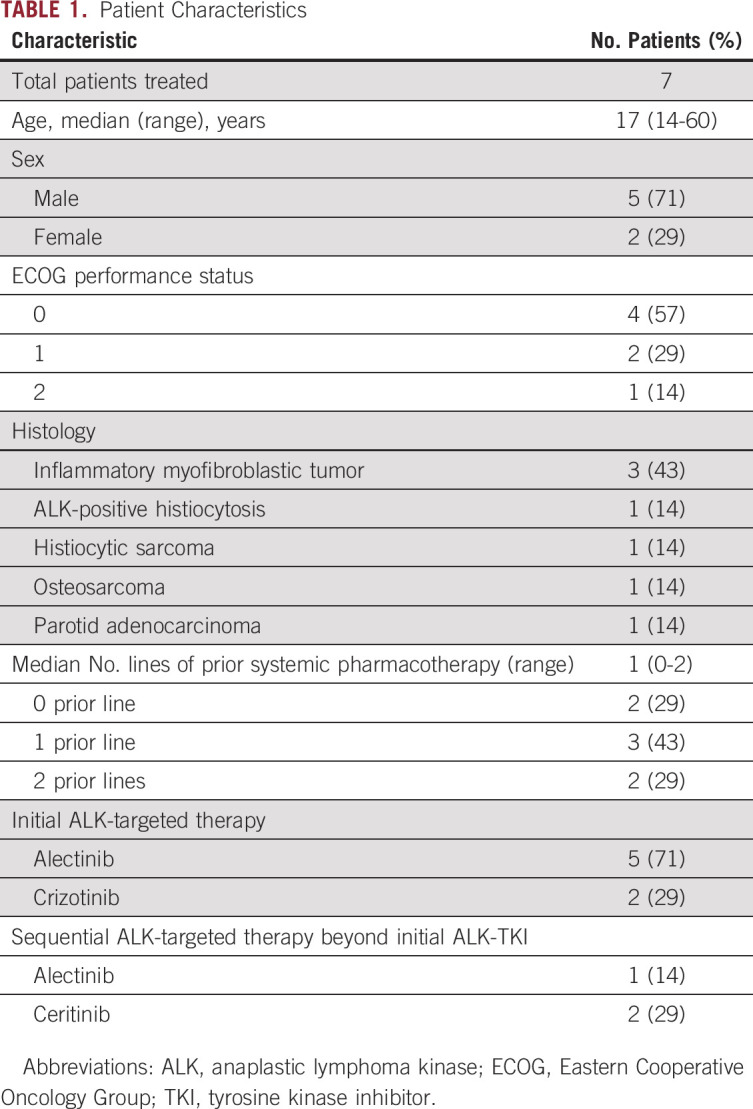
Patient Characteristics

**FIG 1.**
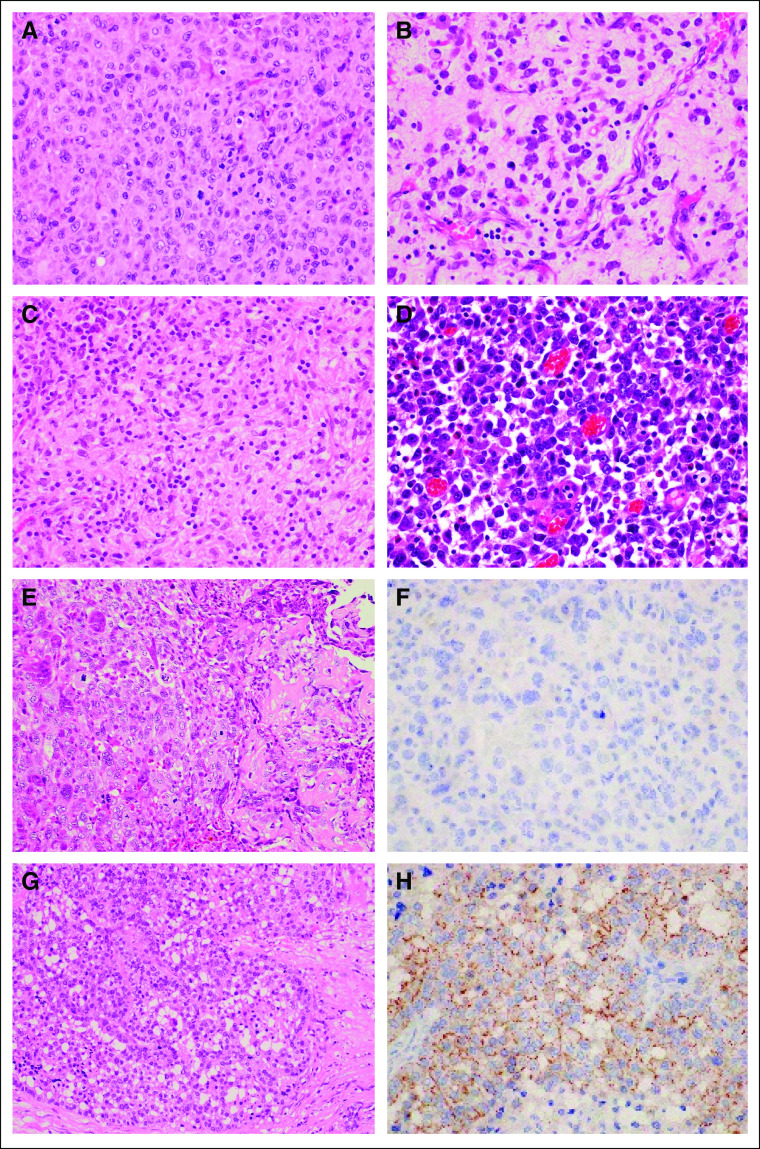
Histological features of representative cases. (A) Patient 1: histiocytic sarcoma, *CLTC|ALK* fusion; (B) patient 3: IMT (epithelioid); (C) patient 4: ALK-positive histiocytosis; (D) patient 5: IMT, *CLTC|ALK* fusion; (E) patient 6: osteosarcoma, *ITSN2|ALK* fusion; (F) patient 6: negative staining in ALK IHC; (G) patient 7: parotid adenocarcinoma, *CTNNA1|ALK* fusion; and (H) patient 7: plasma membrane staining in ALK IHC. ALK, anaplastic lymphoma kinase; IHC, immunohistochemistry; IMT, inflammatory myofibroblastic tumor.

There were five male and two female patients, and the mean age was 17 years (range, 14-60 years). The most common histology was IMT (n = 3), followed by ALK-positive histiocytosis (n = 1), histiocytic sarcoma (n = 1), osteosarcoma (n = 1), and parotid adenocarcinoma (n = 1). Three IMTs showed characteristic histology, including two epithelioid variants. In contrast to ALK-positive histiocytosis, the histiocytic sarcoma showed nuclear atypia and high mitotic activity with atypical mitoses. Osteosarcomas were of the conventional osteoblastic type with highly pleomorphic nuclei. One adenocarcinoma of the parotid gland showed a solid pattern without mucous secretion, and IHC was positive for S100, SOX10, and DOG1 and negative for NR4A3. IHC of ALK was positive for all tumors except osteosarcoma. The ALK staining patterns were nuclear membranous in patient 3 with IMT (epithelioid), plasma membranous in patient 7 with parotid adenocarcinoma, and cytoplasmic in the remaining patients (Table 2 and Fig 1).

**TABLE 2.**
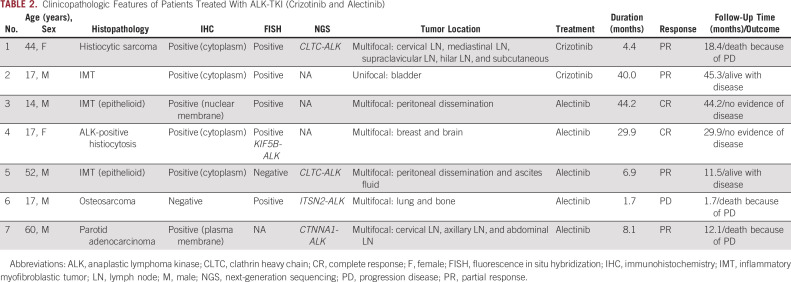
Clinicopathologic Features of Patients Treated With ALK-TKI (Crizotinib and Alectinib)

**FIG 2.**
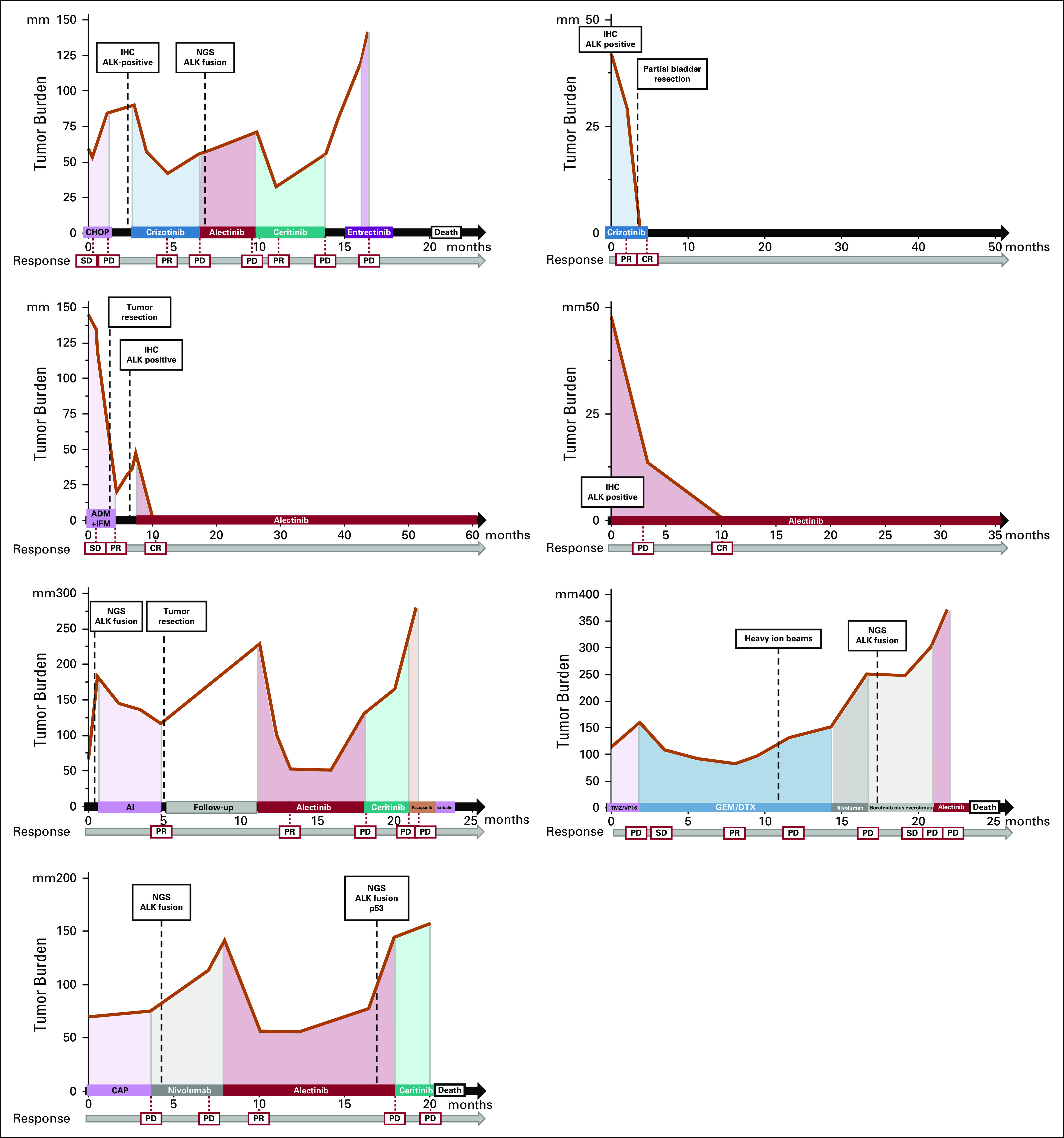
Trends with tumor burden (sum of target lesions) and clinical courses for each case. ADM, adriamycin; AI, adriamycin/ifosfamide; ALK, anaplastic lymphoma kinase; CAP, cyclophosphamide/adriamycin/cisplatin; CHOP, cyclophosphamide/adriamycin/oncovin/prednisolone; CR, complete response; DTX, docetaxel; GEM, gemcitabine; IFM, ifosfamide; IHC, immunohistochemistry; NGS, next-generation sequencing; PD, progressive disease; PR, partial response; SD, stable disease; TMZ, temozolomide; VP-16, etoposide.

**FIG A1.**
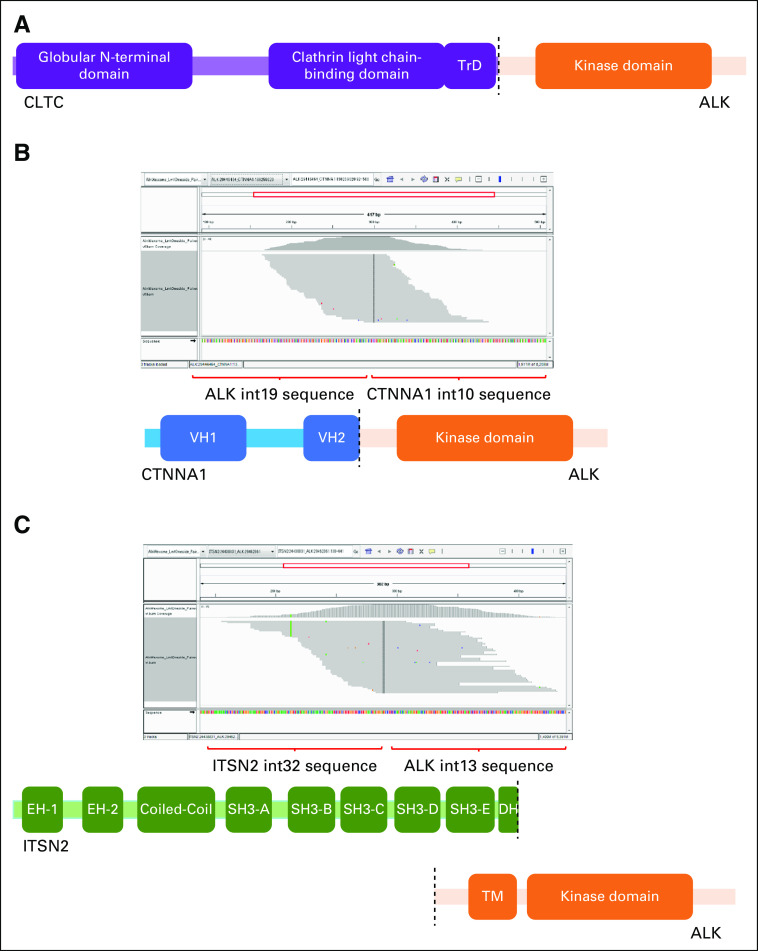
(A) Schematic figures of gene fusions detected by NGS (patients 1 and 5): patient 1: *CLTC*|*ALK* fusion chr17:57, 769, 218-chr2:29, 447, 574 (exon 31: exon 20) and patient 5: *CLTC*|*ALK* fusion chr 17:57, 768, 627-chr 2:29,446,555 (exon 31: exon 20); (B) NGS sequencing results and schematic figure of patient 7: *CTNNA1-ALK* fusion. Patient 7: *CTNNA1*|*ALK* fusion chr5:138, 259, 019-chr2:29, 446, 464 (exon 10: exon 20); (C) NGS sequencing results and schematic figure of patient 6: *ITSN-ALK* fusion. Patient 6: ITSN2|ALK fusion chr2:29,462,361-chr2:24,438,831 (exon 14: exon 32); ALK, anaplastic lymphoma kinase; chr2, chromosome 2; DH, Dbl homology domain; EH, Eps15 homology domain; IMT, inflammatory myofibroblastic tumor; ITSN2, intersectin 2; NGS, next-generation sequencing; SH3, Src three homology domain; TM, transmembrane domain; TrD, trimerization domain; VH1, vinculin homology one domain; VH2, vinculin homology two domain.

The median number of lines of previous systemic pharmacotherapy was one (range, 0-2 lines). All seven patients showed an *ALK* rearrangement of some kind, and four patients were tested by NGS. Their clinicopathological features and detected fusions are listed in Table 2. The observed partner genes were *KIF5B* (n = 1), *CLTC* (n = 2), *ITSN2* (n = 1), and *CTNNA* (n = 1) (Appendix Figs A1A-C). Table 2 and Figure 2 illustrate the patients' clinical courses. Three patients died of cancer, one was lost to follow-up, and the remainder were still alive at last follow-up.

**FIG 3.**
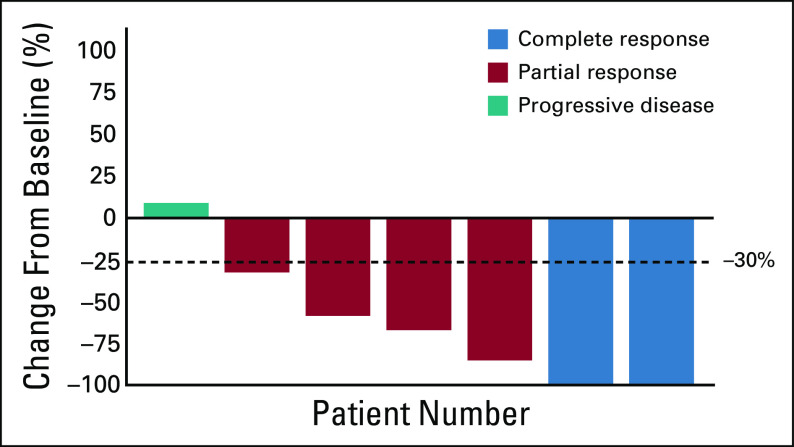
Waterfall plot of best response to initial ALK-TKI. ALK, anaplastic lymphoma kinase; TKI, tyrosine kinase inhibitor.

**FIG 4.**
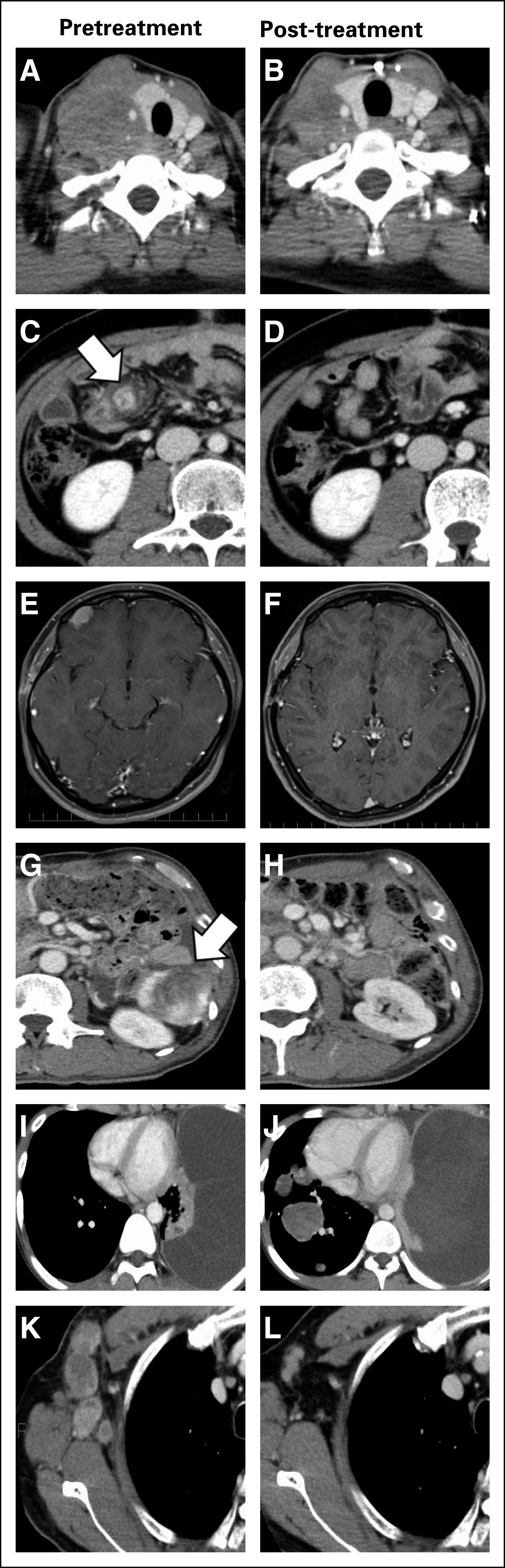
Diagnostic radiographic images of representative cases. (A and B) Patient 1: histiocytic sarcoma showing PR; (C and D) patient 3: IMT (epithelioid) showing CR; (E and F) patient 4: ALK-positive histiocytosis showing CR; (G and H) patient 5: IMT; (I and J) patient 6: osteosarcoma showing PD; and (K and L) patient 7: parotid adenocarcinoma showing PR. ALK, anaplastic lymphoma kinase; CR, complete response; PD, progressive disease; PR, partial response; IMT, inflammatory myofibroblastic tumor.

The best objective response rate (ORR) for initial ALK-TKI was 85.7% (95% CI, 43.65 to 96.99) (6 of 7 patients) with a disease control rate of 85.7% (6 of 7 patients), as summarized in Figure 3. The median progression-free survival (PFS) was 8.1 months (range, 1.7 to not estimable). In patients receiving initial alectinib, the response rate was 80.0% (4 of 5 patients), including two patients with CR and another two with durable PR (Fig 4). In one 17-year-old patient with locally advanced bladder IMT (patient 2 in Table 2), it was possible to preserve the bladder because of the good response to crizotinib.^22^ Three patients were treated with a second ALK-TKI, either alectinib or ceritinib. In one patient initially treated with crizotinib (patient 1), alectinib and ceritinib were subsequently administered. In this patient, alectinib failed to achieve clinical efficacy although ceritinib achieved PR. Overall, the clinical effect of sequential ALK-TKI therapy was mild in terms of the tumor response and short response duration.

Regarding the safety profile of ALK-TKI treatment, there were no treatment-related adverse events and no dose reductions or interruptions for any cause with alectinib therapy. One patient receiving crizotinib experienced grade 3 neutropenia that was considered to be drug related, and dose reduction was required.

## DISCUSSION

The current case series clarified the efficacy of ALK-TKIs, including alectinib, a second-generation ALK-TKI, across different tumor types and fusion partners in patients with advanced, *ALK*-rearranged, nonlung solid tumors. For the first time, we report the response to ALK-TKI in tumors with two rare fusions, *ITSN2-ALK* and *CTNNA1-AL*K, and the clinical benefit and safety of alectinib in a pediatric patient with a solid tumor. Previous studies have shown that crizotinib, a first-generation ALK-TKI, is effective and achieves a durable response in ALK-positive tumors, excluding NSCLC.^10,23,24^ In these studies, crizotinib resulted in ORRs of 66.7%-86.0% in patients with IMT and 11.8% in patients with other solid tumors excluding NSCLC. As for alectinib, a phase II trial for ALK-positive ALCL^25^ led to regulatory approval in Japan, but no clinical trial data for alectinib to treat solid tumors have been reported so far. In our report, the ORR for initial ALK-TKI therapy was 85.7% (6 of 7 patients), which is comparable with the efficacy in previous crizotinib trials. Our ORR results are also similar to those from alectinib trials for NSCLC, although the PFS was shorter than that of the patients with NSCLC.^11^ The shorter PFS could be explained by differences in histology, fusion partner genes, number of lines of previous pharmacotherapy, and number of patients. This report is the first to show the efficacy of alectinib as an initial ALK-TKI in a group of nonlung solid tumors, and the first to report the clinical benefit of alectinib in rare cancer types such as *ALK*-positive histiocytosis, histiocytic sarcoma, and parotid gland adenocarcinoma with *CTNNA-ALK*, in addition to tumors with known fusion types such as IMT with *CLTC-ALK* fusion.

The function of the protein derived from *CTNNA1-ALK* fusion was previously unknown, since only one study described *CTNNA1* as a fusion partner of *ALK* in a patient with salivary secretory carcinoma, and the treatment outcomes were not reported.^26^ Our case of *CTNNA-ALK*–positive parotid adenocarcinoma demonstrated rearrangement in the canonical exon 20 recombination region and showed a clinical response to alectinib (Appendix Fig A1B and Table 2); this was consistent with the other cancer types in our series that had an *ALK* fusion in the same region and also demonstrated a response to alectinib. Also, previous studies have reported that this type of in-frame fusion to exon 20 of *ALK* generates an oncogenic protein, which suggests that this oncogenic protein is a possible therapeutic target of ALK inhibitors irrespective of cancer type.^4,8,27,28^ The positive ALK IHC staining in the tumor cells of our patient with parotid adenocarcinoma and the response to ALK inhibitors suggest that this fusion was an oncogenic driver. Interestingly, ALK plasma membrane staining was characteristic of this patient, which suggests that this staining pattern may reflect CTNNA1 function and location. α-E-catenin, the protein product of *CTNNA1*, functions in a complex with β-catenin and is responsible for organizing and tethering actin filaments at the zones of E-cadherin–mediated cell-cell contact, which can be seen in the cell membrane when stained by IHC for α-E-catenin.^29,30^ Different ALK staining patterns have been described in *ALK*-rearranged tumors depending on the localization of the various fusion proteins.^31^

We also detected an *ITSN2-ALK* fusion in a patient with osteosarcoma who demonstrated poor sensitivity to alectinib. Although many previous studies have reported other *ALK* fusion partners and breakpoints, little is still known about the true oncogenic role of fusion variants other than the common fusions found in NSCLC,^32^ let alone the clinical efficacy of ALK-TKIs for rare *ALK* fusion variants. The *ITSN2-ALK* fusion gene we identified by NGS resulted from a fusion between *ITSN2* (exon 32) and *ALK* (exon 14). However, IHC was negative despite FISH positivity with predominant isolated *ALK* 3′ signals, suggesting that this fusion may not be an activating alteration despite genomic rearrangement. Although our NGS analysis detected *ALK-ITSN2* fusion reads, that is, the possible reciprocal counterpart of *ITSN2-ALK*, it is possible that an *ITSN2-ALK* gene fusion also occurs. Since the fusion gene maintained the ALK kinase domain and the *ITSN2* portion of the fusion gene included the coiled coil domain, the rearranged gene might have resulted in production of an oncogenic fusion protein. However, given the negative IHC results and the poor response to alectinib, the rearrangement is unlikely to have produced the *ITSN2-ALK* fusion gene (Appendix Fig A1C). Only one other *ITSN2* (exon 29)-*ALK* (exon 18) fusion has been reported so far, specifically in a patient with thyroid cancer.^33^ In that case, RNA-Seq showed overexpression of *ALK* exons 18-29 downstream of the fusion point; however, neither IHC results nor the therapeutic effects of ALK-TKIs were reported.

Next-generation ALK-TKIs such as alectinib, ceritinib, and lorlatinib have shown antitumor activity in patients with ALK-positive NSCLC who were previously treated with a different ALK-TKI.^34,35^ In our study, two patients (one with *CLTC-ALK* and the other with *CTNAA-ALK*) who progressed on alectinib were treated with ceritinib. One patient (*CLTC-ALK*) achieved PR, confirming the clinical efficacy of ceritinib. This patient had initially responded to crizotinib, but did not show a response to subsequent alectinib. Since a previous study found that ceritinib was effective in patients with NSCLC treated with first-line alectinib, our results are in line with expectations about rechallenging with ALK-TKIs.^34^ We could not examine the molecular mechanism of resistance to previous ALK-TKI treatment or the efficacy of lorlatinib, a third-generation ALK-TKI. Resistance to ALK inhibitors in *ALK*-rearranged NSCLC is known to result from secondary mutations such as gatekeeper mutations or the emergence of fusion-negative tumor cells.^36-38^ Therefore, we conducted plasma NGS (Guardant360) in the patient with *CTNNA1-ALK* fusion–positive parotid adenocarcinoma at disease progression after he was treated with alectinib. We identified *TP53* T253A and *PIK3CA* E547A mutations but did not detect any *ALK*-related alterations, including the *ALK* fusion found in the tumor tissue. The failure to identify this *ALK* fusion in the serial biopsy may be a result of the alectinib treatment or the limited detection power of NGS when using cell-free DNA. Moreover, Petros et al reported that detection of *TP53* mutations was associated with poor TKI-response in patients with ALK-positive NSCLC.^39^ These factors may explain the poor outcome after treatment with ceritinib in our patient with parotid adenocarcinoma.

This case series has several limitations. First, this was a retrospective study conducted at a single institution with a small sample size and a variety of malignant solid tumors. In addition, because of the lack of systematic strategy at the time to identify patients with rare cancer with a specific genomic characteristic, we did not systematically identify ALK-positive solid tumors. Therefore, there might have been ALK-positive cases that did not receive ALK-TKIs outside of this case series. However, these study characteristics are not unusual because of the nature of rare cancers and we have made improvements in the process by building a registry study for rare cancers. Second, we did not perform NGS sequencing in all patients, and therefore, it was not possible to demonstrate a clear correlation between the efficacy of ALK-TKI treatment and each *ALK* fusion partner. In the era of personalized medicine involving the idea of the $1,000 genome, such precise mechanisms may gradually become clarified, since only FISH was performed before the development of NGS. The MASTER KEY project^40^ is a platform study being conducted in Japan that includes a prospective registry study and multiple clinical trials (UMIN000027552). One of the clinical trials is an investigator-initiated, single-arm, open-label, phase II trial of alectinib for patients with ALK-positive rare cancers (JMA-IIA00364). These platforms are essential since they centrally accumulate limited data in a comprehensive manner, as opposed to instances in which each patient with rare cancer is treated with a driver-directed therapy at a local hospital, and the valuable patient data are scattered.

In conclusion, our data suggest that *ALK* fusions are found in rare solid tumors outside of NSCLC and will lead to clinical benefit for patients in the era of personalized medicine. The ongoing clinical phase II trial is expected to result in new evidence and treatment options for this small patient population.

## References

[1] Morris SW Kirstein MN Valentine MB et al Fusion of a kinase gene, ALK, to a nucleolar protein gene, NPM, in non-Hodgkin's lymphoma Science 263 1281–1284 1994 8122112 10.1126/science.8122112

[2] Griffin CA Hawkins AL Dvorak C et al Recurrent involvement of 2p23 in inflammatory myofibroblastic tumors Cancer Res 59 2776–2780 1999 10383129

[3] Lawrence B Perez-Atayde A Hibbard MK et al TPM3-ALK and TPM4-ALK oncogenes in inflammatory myofibroblastic tumors Am J Pathol 157 377–384 2000 10934142 10.1016/S0002-9440(10)64550-6 PMC1850130

[4] Soda M Choi YL Enomoto M et al Identification of the transforming EML4-ALK fusion gene in non-small-cell lung cancer Nature 448 561–566 2007 17625570 10.1038/nature05945

[5] Pillay K Govender D Chetty R ALK protein expression in rhabdomyosarcomas Histopathology 41 461–467 2002 12405914 10.1046/j.1365-2559.2002.01534.x

[6] Murugan AK Xing M Anaplastic thyroid cancers harbor novel oncogenic mutations of the ALK gene Cancer Res 71 4403–4411 2011 21596819 10.1158/0008-5472.CAN-10-4041 PMC3129369

[7] Molenaar JJ Koster J Zwijnenburg DA et al Sequencing of neuroblastoma identifies chromothripsis and defects in neuritogenesis genes Nature 483 589–593 2012 22367537 10.1038/nature10910

[8] Mano H ALKoma: A cancer subtype with a shared target Cancer Discov 2 495–502 2012 22614325 10.1158/2159-8290.CD-12-0009

[9] Ross JS Ali SM Fasan O et al ALK fusions in a wide variety of tumor types respond to anti-ALK targeted therapy Oncologist 22 1444–1450 2017 29079636 10.1634/theoncologist.2016-0488 PMC5728036

[10] Gambacorti-Passerini C Orlov S Zhang L et al Long-term effects of crizotinib in ALK-positive tumors (excluding NSCLC): A phase 1b open-label study Am J Hematol 93 607–614 2018 29352732 10.1002/ajh.25043 PMC5947833

[11] Peters S Camidge DR Shaw AT et al Alectinib versus crizotinib in untreated ALK-positive non-small-cell lung cancer N Engl J Med 377 829–838 2017 28586279 10.1056/NEJMoa1704795

[12] Camidge DR Kim HR Ahn MJ et al Brigatinib versus crizotinib in ALK-positive non-small-cell lung cancer N Engl J Med 379 2027–2039 2018 30280657 10.1056/NEJMoa1810171

[13] Soria JC Tan DSW Chiari R et al First-line ceritinib versus platinum-based chemotherapy in advanced ALK-rearranged non-small-cell lung cancer (ASCEND-4): A randomised, open-label, phase 3 study Lancet 389 917–929 2017 28126333 10.1016/S0140-6736(17)30123-X

[14] Solomon BJ Besse B Bauer TM et al Lorlatinib in patients with ALK-positive non-small-cell lung cancer: Results from a global phase 2 study Lancet Oncol 19 1654–1667 2018 30413378 10.1016/S1470-2045(18)30649-1

[15] Schöffski P Sufliarsky J Gelderblom H et al Crizotinib in patients with advanced, inoperable inflammatory myofibroblastic tumours with and without anaplastic lymphoma kinase gene alterations (European Organisation for Research and Treatment of Cancer 90101 CREATE): A multicentre, single-drug, prospective, non-randomised phase 2 trial Lancet Respir Med 6 431–441 2018 29669701 10.1016/S2213-2600(18)30116-4

[16] Saiki M Ohyanagi F Ariyasu R et al Dramatic response to alectinib in inflammatory myofibroblastic tumor with anaplastic lymphoma kinase fusion gene Jpn J Clin Oncol 47 1189–1192 2017 28977547 10.1093/jjco/hyx133

[17] Pal SK Bergerot P Dizman N et al Responses to alectinib in ALK-rearranged papillary renal cell carcinoma Eur Urol 74 124–128 2018 29685646 10.1016/j.eururo.2018.03.032

[18] Rao N Iwenofu H Tang B et al Inflammatory myofibroblastic tumor driven by novel NUMA1-ALK fusion responds to ALK inhibition J Natl Compr Canc Netw 16 115–121 2018 29439172 10.6004/jnccn.2017.7031

[19] Honda K Kadowaki S Kato K et al Durable response to the ALK inhibitor alectinib in inflammatory myofibroblastic tumor of the head and neck with a novel SQSTM1-ALK fusion: A case report Invest New Drugs 37 791–795 2019 30790150 10.1007/s10637-019-00742-2

[20] Sunami K Ichikawa H Kubo T et al Feasibility and utility of a panel testing for 114 cancer-associated genes in a clinical setting: A hospital-based study Cancer Sci 110 1480–1490 2019 30742731 10.1111/cas.13969 PMC6447843

[21] Eisenhauer EA Therasse P Bogaerts J et al New response evaluation criteria in solid tumours: Revised RECIST guideline (version 1.1) Eur J Cancer 45 228–247 2009 19097774 10.1016/j.ejca.2008.10.026

[22] Nagumo Y Maejima A Toyoshima Y et al Neoadjuvant crizotinib in ALK-rearranged inflammatory myofibroblastic tumor of the urinary bladder: A case report Int J Surg Case Rep 48 1–4 2018 29758320 10.1016/j.ijscr.2018.04.027 PMC6019858

[23] Mossé YP Lim MS Voss SD et al Safety and activity of crizotinib for paediatric patients with refractory solid tumours or anaplastic large-cell lymphoma: A Children's Oncology Group phase 1 consortium study Lancet Oncol 14 472–480 2013 23598171 10.1016/S1470-2045(13)70095-0 PMC3730818

[24] Mossé YP Voss SD Lim MS et al Targeting ALK with crizotinib in pediatric anaplastic large cell lymphoma and inflammatory myofibroblastic tumor: A Children's Oncology Group study J Clin Oncol 35 3215–3221 2017 28787259 10.1200/JCO.2017.73.4830 PMC5617123

[25] Nagai H Fukano R Sekimizu M et al Phase II trial of CH5424802 (alectinib hydrochloride) for recurrent or refractory ALK-positive anaplastic large cell lymphoma: Study protocol for a non-randomized non-controlled trial Nagoya J Med Sci 79 407–413 2017 28878445 10.18999/nagjms.79.3.407 PMC5577026

[26] Sasaki E Masago K Fujita S et al Salivary secretory carcinoma harboring a novel ALK fusion: Expanding the molecular characterization of carcinomas beyond the ETV6 gene Am J Surg Pathol 44 962–969 2020 32205481 10.1097/PAS.0000000000001471

[27] Sasaki T Rodig SJ Chirieac LR et al The biology and treatment of EML4-ALK non-small cell lung cancer Eur J Cancer 46 1773–1780 2010 20418096 10.1016/j.ejca.2010.04.002 PMC2888755

[28] Yakirevich E Resnick MB Mangray S et al Oncogenic ALK fusion in rare and aggressive subtype of colorectal adenocarcinoma as a potential therapeutic target Clin Cancer Res 22 3831–3840 2016 26933125 10.1158/1078-0432.CCR-15-3000

[29] Rimm DL Koslov ER Kebriaei P et al Alpha 1(E)-catenin is an actin-binding and -bundling protein mediating the attachment of F-actin to the membrane adhesion complex Proc Natl Acad Sci U S A 92 8813–8817 1995 7568023 10.1073/pnas.92.19.8813 PMC41057

[30] Majewski IJ Kluijt I Cats A et al An α-E-catenin (CTNNA1) mutation in hereditary diffuse gastric cancer J Pathol 229 621–629 2013 23208944 10.1002/path.4152

[31] Pulford K Lamant L Morris SW et al Detection of anaplastic lymphoma kinase (ALK) and nucleolar protein nucleophosmin (NPM)-ALK proteins in normal and neoplastic cells with the monoclonal antibody ALK1 Blood 89 1394–1404 1997 9028963

[32] Rosenbaum JN Bloom R Forys JT et al Genomic heterogeneity of ALK fusion breakpoints in non-small-cell lung cancer Mod Pathol 31 791–808 2018 29327716 10.1038/modpathol.2017.181

[33] Panebianco F Nikitski AV Nikiforova MN et al Characterization of thyroid cancer driven by known and novel ALK fusions Endocr Relat Cancer 26 803–814 2019 31539879 10.1530/ERC-19-0325 PMC7002208

[34] Hida T Seto T Horinouchi H et al Phase II study of ceritinib in alectinib-pretreated patients with anaplastic lymphoma kinase-rearranged metastatic non-small-cell lung cancer in Japan: ASCEND-9 Cancer Sci 109 2863–2872 2018 29959809 10.1111/cas.13721 PMC6125456

[35] Gadgeel SM Gandhi L Riely GJ et al Safety and activity of alectinib against systemic disease and brain metastases in patients with crizotinib-resistant ALK-rearranged non-small-cell lung cancer (AF-002JG): Results from the dose-finding portion of a phase 1/2 study Lancet Oncol 15 1119–1128 2014 25153538 10.1016/S1470-2045(14)70362-6

[36] Choi YL Soda M Yamashita Y et al EML4-ALK mutations in lung cancer that confer resistance to ALK inhibitors N Engl J Med 363 1734–1739 2010 20979473 10.1056/NEJMoa1007478

[37] Katayama R Friboulet L Koike S et al Two novel ALK mutations mediate acquired resistance to the next-generation ALK inhibitor alectinib Clin Cancer Res 20 5686–5696 2014 25228534 10.1158/1078-0432.CCR-14-1511 PMC4233168

[38] Katayama R, Shaw AT, Khan TM (2012). Mechanisms of acquired crizotinib resistance in ALK-rearranged lung Cancers. Sci Transl Med.

[39] Christopoulos P, Dietz S, Kirchner M (2019). Detection of TP53 mutations in tissue or liquid rebiopsies at progression identifies ALK+ lung cancer patients with poor survival. Cancers (Basel).

[40] Okuma HS Yonemori K Narita SN et al MASTER KEY project: Powering clinical development for rare cancers through a platform trial Clin Pharmacol Ther 108 596–605 2020 32112563 10.1002/cpt.1817 PMC7484913

